# New approaches to the treatment of metabolic dysfunction-associated steatotic liver with natural products

**DOI:** 10.1016/j.iliver.2024.100131

**Published:** 2024-11-08

**Authors:** Pooja Yadav, Khushi Quadri, Renu Kadian, Aafrin Waziri, Pankaj Agrawal, Md Sabir Alam

**Affiliations:** a Department of Pharmacy Practice, SGT College of Pharmacy, SGT University, Gurgaon-Badli Road Chandu, Gurugram 122505, India; b Department of Pharmaceutics, SGT College of Pharmacy, SGT University, Gurgaon-Badli Road Chandu, Gurugram 122505, India; c Ram Gopal College of Pharmacy, Gurugram University, Gurugram 122506, India; d University School of Biotechnology, Guru Gobind Singh Indraprastha University, Dwarka Delhi 110078, India; e Pharmaceutical Sciences and University School of Medicine and Allied Sciences, Guru Gobind Singh Indraprastha University, Dwarka Delhi 110078, India

**Keywords:** MASLD, Natural products, Alcohol consumption, Liver disorder, Therapy

## Abstract

Metabolic dysfunction-associated steatotic liver disease (MASLD) is a widespread and potentially severe liver condition characterized by abnormal fat accumulation in the liver unrelated to alcohol consumption. According to the World Health Organization, MASLD is the most prevalent liver disease globally, it affects approximately 25% of the world’s population, nearly two billion individuals. This staggering prevalence underscores the urgent need for effective and safe therapeutic approaches to address this escalating global health concern. Natural products have emerged as promising candidates for preventing and treating MASLD in recent years because of their diverse bioactive compounds and minimal side effects. Well-known natural products such as curcumin, resveratrol, green tea polyphenols, and silymarin exhibit notable hepatoprotective effects by influencing lipid metabolism, mitigating oxidative stress, and reducing inflammation. Ongoing research highlights the potential of phytochemicals from traditional medicinal plants, such as *Phyllanthus* and *Salvia*, in ameliorating liver steatosis and fibrosis. These natural products demonstrate the capacity to impede fibrogenesis by interfering with hepatic stellate cell activation, which is pivotal in liver fibrosis development. Recent studies underscore the significance of natural products in modulating the gut–liver axis, where they restore balance to the gut microbiota and enhance intestinal barrier function, which slows the progression of MASLD. Moreover, advancements in nanotechnology facilitate the targeted delivery of natural product-derived compounds, which enhances their bioavailability and therapeutic efficacy. Harnessing the potency of natural products offers a promising avenue for developing novel, safer therapies for MASLD and addressing a critical global health concern with far-reaching implications for public well-being.

## Graphical abstract

**Graphic Abstract:** The role of natural compounds in treating MAFLD disease via different mechanistic approaches. **SREBP-1c:** Sterol regulatory element-binding protein-1c, **Nrf2:** Nuclear factor-erythroid 2-related factor 2, **PPAR:** Peroxisome proliferator-activated receptor, **AMPK:** AMP-activated protein kinase, **SIRT:** Sirtuin, **NLRP3:** NOD-like receptor family pyrin domain-containing 3, **SAMC:** S-ally mercapto cysteine, **DADS:** Diallyl disulfide, **TLR-4:** Toll like receptor 4, **COX-2:** Cyclooxygenase-2, **EGCG:** Epigallocatechin gallate, **RCTs9:** Randomized Controlled/Clinical Trial, **TGF-β:** transforming growth factor-beta.

**Figure:**
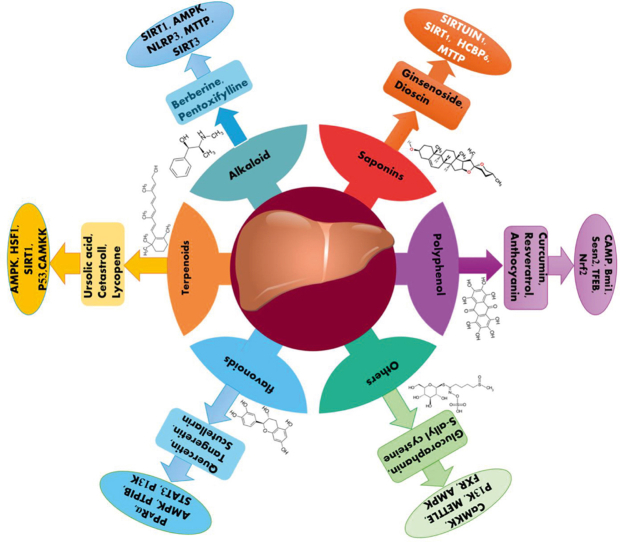

## 1 Introduction

Metabolic dysfunction-associated steatotic liver disease (MASLD) previously known as non-alcoholic fatty liver disease (NAFLD) has emerged as a silent epidemic. MASLD represents a spectrum of liver disorders ranging from simple steatosis to metabolic dysfunction-associated steatohepatitis (MASH) previously known as non-alcoholic steatohepatitis (NASH) [1], fibrosis, cirrhosis [2], and potentially hepatocellular carcinoma [3]. Characterized by excessive hepatic fat accumulation in individuals without a history of significant alcohol consumption [4], MASLD is closely associated with obesity, insulin resistance, and metabolic syndrome [5]. According to the World Health Organization, the global prevalence of MASLD has reached alarming levels: it now affects approximately 25% of the world’s population (almost two billion people), making it one of the most common liver diseases worldwide [6]. Traditionally, MASLD management has focused on lifestyle modifications, including dietary changes and increased physical activity [7]. However, the intricate interplay of genetic [8], environmental [9], and metabolic factors that contribute to MASLD necessitates innovative and targeted therapeutic approaches [10]. In recent years, natural products have emerged as promising candidates for MASLD treatment and prevention because of their rich reservoir of bioactive compounds including polyphenols [11], flavonoids [12], terpenoids [13], and alkaloids [14]. These compounds, derived from plants, herbs, and traditional medicinal sources, show remarkable potential in combating various aspects of MASLD pathogenesis and offer new horizons for liver health [15]. The appeal of natural products in MASLD management lies in their multifaceted mechanisms of action. These compounds exhibit antioxidant [16], anti-inflammatory [17], anti-fibrotic [18], and anti-lipogenic properties [19]; they therefore target key pathways involved in MASLD progression. Moreover, their relatively low toxicity and minimal side effects make them attractive candidates for long-term therapeutic interventions [20].

### 1.1 Bioactive compounds

Natural products contain a wide range of bioactive compounds that have significant hepatoprotective effects [21]. Curcumin [22], derived from turmeric, has been extensively studied for its anti-inflammatory and antioxidant properties [23]. It attenuates hepatic steatosis by regulating lipid metabolism and modulating inflammatory pathways [24]. Resveratrol [25], found in grapes and berries, shows promise in reducing hepatic fat accumulation, oxidative stress, and inflammation, making it a potential therapeutic agent for MASLD [26]. Green tea polyphenols [27], particularly epigallocatechin gallate, exert anti-lipogenic effects by inhibiting lipid absorption and promoting fat oxidation. Additionally, silymarin [28], a flavonoid extracted from milk thistle, exerts hepatoprotective properties by stabilizing cell membranes, inhibiting oxidative stress, and modulating inflammatory responses [29].

#### 1.1.1 Gut–Liver Axis Modulation

Recent research has emphasized the critical role of the gut–liver axis in MASLD pathogenesis [30]. Disruptions in gut microbiota composition, termed dysbiosis, contribute to increased intestinal permeability and bacterial translocation, which triggers hepatic inflammation and fibrosis [31]. Natural products such as probiotics, prebiotics, and dietary fiber act as modulators of gut microbiota; these products restore microbial balance and enhance intestinal barrier function [32]. Probiotics [33], which are live beneficial bacteria, promote gut health by restoring microbial diversity, reducing gut permeability, and mitigating inflammation [34]. Prebiotics [35] are non-digestible fibers that promote the growth of beneficial bacteria, enhance gut integrity, and attenuate hepatic lipid accumulation [36]. The synergistic effects of natural products on the gut–liver axis present a novel therapeutic approach and emphasize the interconnectedness of the gastrointestinal tract and liver health [37].

#### 1.1.2 Nanotechnology (enhancing natural product efficacy)

Advancements in nanotechnology have transformed the treatment of MASLD by enhancing the efficacy of natural products [38]. Nanoparticles, liposomes, and micelles are employed to encapsulate bioactive compounds derived from natural products; this protects them from degradation and facilitates targeted delivery to liver cells [39]. These innovative nanostructures improve natural products’ solubility, stability, and absorption, which ensures optimal therapeutic effects [40]. Controlled release mechanisms prolong the presence of bioactive compounds in the bloodstream, while ligand-functionalized nanoparticles enhance cellular uptake [41], which maximizes their therapeutic potential [42]. Natural products, given their diverse bioactive compounds and minimal side effects, present a promising avenue for MASLD treatment [43,44] and signal a paradigm shift in liver health intervention on a global scale [45,46]. In this review, we discuss the role of various natural products in the treatment of MASLD along with approaches based on nanotechnology including metallic nanoparticles, polymeric nanoparticles, and nanostructured lipid carriers [[47], [48], [49], [50], [51], [52], [53], [54], [55], [56], [57], [58], [59]].

#### 1.1.3 Literature review

The review utilized electronic databases including Science Direct, PubMed, and Google Scholar and employed keywords such as “MASLD” “Natural Products” “Alcohol Consumption” “Liver disorder” and “Therapy” Relevant high-impact publications from January 2016 to August 2023 were selected, excluding unpublished data and conference papers. Only English-language articles were considered. Cross-checking verified the scientific names and types of natural products for treating MASLD.

### 1.2 Treatment of MASLD

#### 1.2.1 By available medicine

Fatty liver disease, also known as MASLD, is a common condition characterized by fat accumulation in the liver. It can range from simple steatosis (fat accumulation) to MASH, which involves inflammation and liver damage. While no specific medication is approved for the treatment of MASLD or MASH till January 2022, several approaches have been explored, including the use of natural products and lifestyle modifications. Here is a detailed explanation of the available treatment options [60].

#### 1.2.2 Lifestyle modifications

##### 1.2.2.1 Weight management

Obesity is a significant risk factor for MASLD. Weight loss through a balanced diet and regular physical activity is the most effective way to improve fatty liver. Even a modest reduction in body weight (5%–10%) can significantly improve liver function [61].

##### 1.2.2.2 Dietary changes

A healthy diet is crucial for managing fatty liver disease. A balanced diet should focus on reducing the intake of saturated and trans fats, refined sugars, and processed foods. A healthy diet emphasizes the consumption of fruits, vegetables, whole grains, lean proteins, and foods high in omega-3 fatty acids, such as fatty fish [60,61].

##### 1.2.2.3 Physical activity

Regular exercise can help to reduce fat accumulation in the liver. The current recommendations are to aim for at least 150 min of moderate-intensity aerobic exercise per week combined with strength training exercises [62].

### 1.3 Phytopharmaceuticals and other supplements

While there are no specific medications approved for MASLD or MASH, some natural products and dietary supplements are beneficial in managing the condition. Consultation with a healthcare provider before taking any supplements is essential, as they may have interactions or side effects. Some examples are listed below.

#### 1.3.1 Omega-3 fatty acids

Fish oil supplements, rich in omega-3 fatty acids, may help to reduce liver fat and inflammation [61].

#### 1.3.2 Vitamin E

Some studies suggest that vitamin E supplements can benefit individuals with MASH, although a healthcare professional should supervise its use because of potential risks.

#### 1.3.3 Management of comorbidities

Management of conditions like diabetes, high blood pressure, or high cholesterol is essential, as they can exacerbate MASLD.

#### 1.3.4 Avoidance of alcohol and medications that can harm the liver

Alcohol consumption and use of medications that may harm the liver should be avoided or discussed with a healthcare provider.

#### 1.3.5 Regular monitoring

Regular follow-up with a healthcare provider is crucial to monitor the progression of the disease and make necessary adjustments to treatment plans [61,62].

### 1.4 Mechanistic approach for the treatment of MASLD

The onset of MASLD is linked to several related key pathological events, such as insulin resistance, abnormal lipid metabolism, oxidative stress, inflammation, apoptosis, and fibrosis. When treating MASLD, herbal therapies can significantly improve steatosis and inflammation, by affecting the processes that are involved in inflammation and lipid metabolism [63].

Determining dietary approaches to MASLD prevention and treatment is extremely important, as there are currently no approved therapies for the condition. However, according to data from studies conducted on animals and cells, numerous medications may prevent MASLD [64]. Traditional medicines employ various mechanisms to prevent MASLD. These mechanisms include the following: (1) down-regulating sterol regulatory element-binding protein-1c (SREBP-1c) expression to depress lipogenesis; (2) up-regulating peroxisome proliferator-activated receptor (PPAR)α expression to increase β-fatty acid (FA) oxidation; (3) up-regulating nuclear factor-erythroid 2-related factor 2 (Nrf2) expression to increase antioxidant levels and depress oxidative stress; and (4) inhibiting the activation of inflammatory pathways. According to recent discoveries, the common trigger regulating these molecular processes is the activation of the AMP-activated protein kinase/sirtuin-1 (AMPK/SIRT-1) signaling pathway. Furthermore, traditional Chinese medicines’ indirect anti-inflammatory and antioxidative properties might ease MASLD symptoms [64,65]. Several natural items have demonstrated efficacy in the treatment of MASLD.

#### 1.4.1 Ginger

Also known as *Zingiber officinale* Roscoe, which belongs to the family Zingiberaceae, ginger is a flowering plant whose rhizome is extensively used in traditional medicine and spices. These rhizomes are harvested, dried, and used for various culinary and medicinal purposes. The active chemical constituent in ginger for the treatment of MASLD is gingerol. Ginger may help treat MASLD by lowering oxidative stress and inflammation; studies using fibro-scan have also show that ginger improves fatty liver scores and liver biomarkers [66,67]. Moreover, ginger may help adipocytes become more sensitive to insulin PARα and PPARγ is thought to have an impact on the pathogenesis of MASLD by influencing the accumulation of hepatic triglycerides [68].

#### 1.4.2 Berberine

The active compound in berberine, an isoquinoline alkaloid, shows potential for treating MASLD. A quaternary ammonium salt, berberine belongs to the benzylisoquinoline alkaloids’ protoberberine group. It can be found in the stem bark, rhizomes, and roots of many plants, such as tree turmeric, barberry, and goldenseal. Berberine can impact MASLD by lowering insulin resistance, oxidative stress, and inflammation. Berberine also alters the expression of genes and noncoding RNAs, particularly miRNAs, giving it preventive and therapeutic actions against MASLD [69].

One of the primary active metabolites of berberine is berberrubine, which exhibits strong anti-obesity and antihyperglycemic properties. Nevertheless, it is unclear whether berberrubine has an in vivo therapeutic effect on MASLD and what the mechanisms underlying any effects may be [70]. In two unrelated murine models, acetaminophen-induced acute liver injury and methionine and choline-deficient diet-induced MASH, berberine inhibited NOD-like receptor family pyrin domain-containing 3 (NLRP3) inflammasome activation both in vivo and in vitro [71]. Additionally, berberine modulated purinergic receptor P2X, ligand-gated ion channel 7 signalling to counteract NLRP3 inflammasome activation in vitro in lipopolysaccharide-treated macrophages. Berberine also reduced high-fat diet induced insulin resistance in vivo and palmitate-induced NLRP3 inflammasome activation by inducing autophagy in macrophages in vitro [72].

#### 1.4.3 Lychee pulp

Derived from the succulent fruit of the *Litchi chinensis* tree, which harbors a treasure trove of phenolic compounds, lychee pulp is the fruit’s edible flesh. Lychee pulp benefits MASLD by lowering oxidative stress and inflammation and is a rich source of antioxidants. The active chemical constituent in lychee pulp relevant to the treatment of MASLD is quercetin-3-rutinose-7-rhamnoside [68].

#### 1.4.4 Grape seed

Grape seed acts mainly by lowering oxidative stress as these are rich source of antioxidant. The main active chemical constituents of grape seed in the treatment of MASLD are catechin and epicatechin [69].

#### 1.4.5 Rosemary

The main active chemical constituents of rosemary in the treatment of MASLD are rosmarinic acid, carnosic acid, rosmanol, carnosol, and ursolic acid. Also known as *Rosmarinus officinalis*, this fragrant herb is frequently used in cooking. I have antioxidant and anti-inflammatory properties which makes it beneficial in the treatment of MASLD [73,74].

#### 1.4.6 Terpenoids

Terpenoids are naturally occurring substances that exhibit various biological activities and diverse structures and distributions. The main active chemical constituent of terpenoids in the treatment of MASLD is paeoniflorin. Terpenoids are made of isoprene and are crucial to every organism’s metabolism [75]. They also have anti-inflammatory and antioxidant properties, making them promising therapeutic agents for a variety of inflammatory illnesses [76,77]. Numerous terpenoids derived from plants inhibit the activation of the NLRP3 inflammasome, which results in anti-MASH effects. Terpenoids have multiple hydrocarbon isoprene units and oxygenated derivatives in their molecular formulas. These oxygenated derivatives include alcohol, aldehydes, ketones, carboxylic acids, and esters. Terpenoids are abundant in nature and are the primary constituents of pigment resins and certain plant essences [78]; they exhibit a wide range of physiological effects, such as expectoration, cough relief, evaporation, perspiration induction, insecticidal action, and analgesia (pain reduction) [79]. Several plants, particularly *Betula*, contain the pentacyclic triterpene betulinic acid and betulin. Betulin, the precursor of betulinic acid, is convertible to its product. By altering the AMPK-SREBP signaling pathway, betulinic acid significantly reduces hepatic lipid accumulation [80].

#### 1.4.7 Antcin A

Traditional Chinese medicine uses antcin A, a terpenoid isolated from *Antrodia camphorata*, as a treatment for MASLD. Strong hepatoprotective and anti-inflammatory properties are exhibited by antcin A [81]. Research conducted both in vivo and in vitro demonstrates that antcin A can improve hepatic lipid accumulation, lower alanine and aspartate amine transferase levels, and suppress the expression of pyroptosis-associated proteins (the full-length and N-terminal domain of gasdermin D) and the NLRP3 inflammasome (which includes NLRP3, apoptosis-associated speck-like protein containing a CARD, and caspase-1). Antcin A treatment significantly decreases both the number of pyroptotic cells and the pore permeability of the cell membrane by attaching itself to NLRP3 and preventing the NLRP3 inflammasome from assembling and activating, which would otherwise enhance MASH and further mediate the inflammatory response and pyroptosis [82].

#### 1.4.8 Alkaloids

Alkaloids are a class of naturally occurring nitrogenous organic compounds found in large numbers in dicotyledons. The main active chemical constituents of alkaloids in the treatment of MASLD are obeticholic acid, cenicriviroc, and belapectin. Their pharmacological properties include antibacterial, analgesic, anti-inflammatory, anti-tumor, and anti-fungal effects [[83], [84], [85]]. Numerous studies show that alkaloids significantly affect MASLD [[86], [87], [88]].

#### 1.4.9 Coffee

Caffeine, chlorogenic acid, cafestol, and kahweol are just a few of the many bioactive substances found in coffee. The main active chemical constituent of coffee in the treatment of MASLD is caffeic acid (CA) [89]. Certain bioactive compounds, such as trigonelline and caffeic acid, improve liver triglyceride metabolism, oxidative stress, and fibrotic status, all of which are associated with a lower risk of fatty liver disease [90]. By correcting the imbalance in the gut microbiota and the resulting lipopolysaccharide-mediated inflammation, CA prevents the expression of genes linked to lipid metabolism from becoming dysregulated; therefore, CA may be utilized as a therapeutic strategy for MASLD linked to obesity.

#### 1.4.10 Curcumin

Turmeric’s main active ingredient is curcumin, a naturally occurring polyphenol that has been utilized in traditional Chinese medicine and Ayurvedic medicine for many years. Preclinical mouse models show curcumin reduces MASLD [91]. Curcumin reduces MASLD by inhibiting NLRP3 and by modulating oxidative stress, lipid metabolism, AMPK and Nrf2 activation, gut microbiota, to control inflammation and lower hepatic steatosis; however, more research is needed to confirm their benefits in clinical MASLD patients [92].

#### 1.4.11 Silymarin

Silymarin, commonly referred to as milk thistle extract, possesses a combination of components that exhibit strong hepatoprotective properties against various liver diseases [93]. The active ingredients are taxifolin, isosilybin A, silybin A, silybin B, silibinin, and their combinations. Preclinically, silymarin or one or more of its hepatoprotective components alleviates MASH in both C57BL/6J mice fed a methionine and choline-deficient diet and db/db mice [94], as well as in hamsters and mice [95] with high-fat diet-induced MASLD. Silymarin may modify the gut microbiota, which could mediate its effects on metabolic disorders. To treat MASLD or MASH, silymarin impacts several targets including farnesoid X receptor, Nrf2, SIRT1, SIRT2, AMPK, and the NLRP3 inflammasome. Clinical data point to silymarin’s potential, if not definitive, role in treating MASLD or MASH [96].

#### 1.4.12 Saponin

Mostly present in terrestrial plants, saponins are glycoside aglycones of three terpenoids, also known as spirostane compounds [97]. Saponins are the main active components of many traditional Chinese herbs, including *Glycyrrhiza uralensis* (Fisch), *Polygala tenuifolia* (Willd.), *Panax ginseng* (C. A. Mey.), and *Platycodon grandiflorus* (Jacq.) A. DC. Additionally, some saponins have anti-cancer, antibacterial, and antipyretic properties [98,99]. Dioscin, a naturally occurring steroid saponin, is present in many different herbs [100]. Dioscin possesses anti-tumour [101], anti-hyperlipidemic, and anti-fungal properties [102]. Research indicates that dioscin supplementation in mice with obesity can help them lose weight gradually without increasing physical activity or suppressing appetite. By inhibiting fatty acid synthase, oral dioscin administration lowers blood lipid levels; lowers fat and cholesterol accumulation in the liver, and triglyceride deposition; encourages beta-oxidation; and controls the MAPK signalling pathway, autophagy, oxidative stress, and inflammation [103]. Other saponins that are important in the treatment of MASLD include trilling, ginsenoside Rb1, and ginsenoside Rg1 [104].

#### 1.4.13 Resveratrol

In both rodent and cell models, resveratrol, a naturally occurring polyphenol that activates SIRT1, has strong anti-MASLD effects; however, there is still conflicting evidence regarding its impact on individuals with MASLD [105]. A high dose of resveratrol supplementation does not affect body composition, insulin sensitivity, or other inflammatory or metabolic biomarkers, according to a clinical study involving 24 obese but otherwise healthy men [106]. Similarly, studies involving middle-aged, non-obese subjects with normal glucose tolerance did not find any benefit from resveratrol supplements, indicating that resveratrol is ineffective in people without metabolic disease. By contrast, a study of 50 MASLD patients with metabolic disease found that compared with lifestyle changes, 500 mg of resveratrol treatment for 12 weeks improved MASLD [107].

#### 1.4.14 Licorice, Red Clover, and Chamomile

Future research could focus on red clover, chamomile, and licorice, as these herbs are presently being investigated for their potential therapeutic applications. Licorice supports lipid homeostasis via PPARα, carnitine palmitoyltransferase-1α, FA synthase, SREBP-1c, and acetyl-CoA carboxylase 1 [108,109]. In a double-blind clinical trial, 66 patients were randomly assigned to receive 2 g of aqueous licorice root extract or a placebo for two months only the licorice treated group’s aspartate and alanine aminotransferase levels improved [110]. Because of their potential involvement in PPARα regulation, red clover and chamomile may have hepatoprotective effects; however, further research is required to fully understand their effects on MASLD [111,112].

#### 1.4.15 Ginseng

In traditional Chinese medicine, ginseng, or *P. ginseng*, is frequently used to treat a variety of metabolic disorders, including hepatosteatosis [113,114]. Ginsenosides and saponins are the active ingredients in ginseng [115]. By inducing the SIRT1/AMPK pathway and activating autophagocytic pathways, ginseng decreases lipid accumulation [115,116]. Additionally, ginseng inhibits hepatic stellate cell activation and lowers hepatic fibrosis factors like collagen-1 and α-smooth muscle actin [116]. Through glucose-regulated protein 78, ginseng also lessens endoplasmic reticulum stress and inflammation. Supplementing MASLD/MASH-induced mice with ginseng extract improves hepatic inflammation, fibrosis, liver enzymes, and steatosis [115,116]. Therefore, according to available data, ginseng may be a useful compound to investigate in MASLD patients in future studies in Table 1, Table 2, Table 3.

**Table 1:** Treatment of MASLD with natural products.

S. No.	Plant Source	Chemical Constituent	Mechanism	Model	Reference
1.	Ginger	Gingerol	Ginger can inhibit arachidonic acid metabolism with the suppression of cyclooxygenase and lipooxygenase enzymes, thereby acting as an anti-inflammatory agent. Ginger has potential antioxidant activities due to its polyphenolic compounds, such as gingerol.	*In vivo*	[117]
2.	Berberine	Isoquinoline alkaloid	It regulates genes involved in glucose and fatty acid synthesis. Live triglycerides, cholesterol, and body weight.	Animal model	[118,119]
3.	Lychee pulp	Quercetin-3-rutinose-7-rhamnoside	Lowering oxidative stress and inflammation, by altering gene expression & noncoding RNAs, particularly miRNAs.	Aminal model & RCTs	[68]
4.	Grape seed	Catechin, epicatechin	Lowering oxidative stress and inflammation, by altering gene expression & noncoding RNAs, particularly miRNAs.	Animal model	[69]
5.	Rosemary	Rosmarinic acid, carnosic acid, rosmanol, carnosol, Ursolic acid	Lowering oxidative stress and inflammation, by altering gene expression & noncoding RNAs, particularly miRNAs.	Aminal model & RCTs	[68,69]
6.	Terpenoids	Paeoniflorin	By altering the AMPK-SREBP signaling pathway, betulinic acid significantly contributes to reducing hepatic lipid accumulation	Animal model	[80]
7.	Anctin A		By attaching itself to NLRP3 and preventing the NLRP3 inflammasome from assembling and activating, thus enhancing MASH and further mediating the inflammatory response and pyroptosis	*In vivo*, *In vitro*	[82]
8.	Alkaloids	Obeticholic acid, cenicriviroc, belapectin	It protects against hepatic steatosis and other hallmarks of MASLD through modulating the AMPK-SREBP1 axis. it may therefore have a therapeutic potential for MASLD treatment.	Animal model	[120]
9.	Coffee	Caffeic acid	Correcting the imbalance in the gut microbiota and the resulting lipopolysaccharide-mediated inflammation prevents the expression of genes linked to lipid metabolism from becoming dysregulated	*In vivo*	[121]
10.	Curcumin		AMPK pathway: Curcumin regulates this pathway, involved in lipid metabolism and energy balance. It also impacts the transforming growth factor-beta pathway linked to inflammation and fibrosis.	Animal model	[92]
11.	Silymarin	Taxifolin, isosilybin A, silybin A, silybin B, silibinin	Acts as a free radical scavenger and modulates enzymes associated with the development of cellular damage, fibrosis, and cirrhosis. These hepatoprotective effects were observed in clinical studies in patients with alcoholic or non-alcoholic fatty liver disease, including patients with cirrhosis.	*In vivo*	[96]
12.	Saponin	Dioscin	By inhibiting FAS, oral dioscin administration lowers blood lipid levels, enhances fat accumulation in the liver, lowers liver cholesterol, and triglyceride deposition, and encourages beta-oxidation controls the MAPK signalling pathway, autophagy, and oxidative stress and inflammation	Animal model	[103]
13.	Resveratrol		Activation of AMPK and SIRT1 in hepatic cells and anti-oxidant and anti-inflammatory actions may prevent liver damage and may inhibit the progression of MASLD	Animal model and RCTs.	[105]
14.	Milk thistle	Silibinin, Isosilibin	Antioxidative, anti-apoptotic, anti-inflammatory, anti-adipogenic	Experimental and clinical studies (RCTs)	[122]
15.	Liquorice, red clover, and chamomile		Potential involvement in PPARα regulation, and are thought to have hepatoprotective effects	RCTs	[101,102]
16.	Ginseng	Ginsenosides and saponins	Inducing the SIRT1/AMPK pathway and activating auto-phagocytic pathways, ginseng can decrease lipid accumulation.	Clinical study	[115,116]
17.	Fish oils	Eicosapentaenoic acid, Docosahexanoic acid	Improve lipid homeostasis and inflammation through the PPARα, AMPK, and adiponectin	Clinical study	[123]
18.	Garlic	S-allyl cysteine, S-ally mercapto cysteine, diallyl disulfide	Function as a SIRT1 activator, causing the AMPKα pathway to be activated and lowering hepatic lipogenesis and lipotoxicity.	RCT	[124]
19.	Green tea	Epigallocatechin-3-gallate	Reduces hepatic COX2, prostaglandin E2, NF-κB, and toll-like receptor 4, which in turn reduces hepatic inflammation.	*In vivo*	[125,126]
			Additionally controls hepatic lipid homeostasis by modifying the AMPK, SREBP, PPARγ, and mitochondrial complex chain protein pathways.		

**COX-2-**Cyclooxygenase-2, **RCTs-**Randomized Controlled/Clinical Trial, **AMPK**-adenosine monophosphate-activated protein kinase, **SREBP**-Sterol Regulatory Element Binding Protein, **SIRT1**-silent information regulator sirtuin 1, **PPARα**-peroxisome proliferator-activated receptor alpha, **NLRP3**-NOD-like receptor family pyrin domain-containing 3, **MASLD-**Metabolic dysfunction-associated steatotic liver disease, **MASH-**Metabolic dysfunction-associated steatohepatitis.

**Table 2:** Dosages used to treat MASLD with natural products.

S. No.	Herbal Medicine	*In vivo* effects	*In vivo* Dosing	Duration of study
1.	*Zingiber zerumbet* Smith	HFD induced obese mice	100, 200, or 300 mg/kg	8 weeks
2.	Berberine	HFD-induced mice to target site on SIRT1	1.5 g/kg	16 weeks
3.	Curcumin	Apoe KO mice target site on TFEB	20 mg/kg	16 weeks
4.	Alkaloids	MCD-induced mice to target on TXNIP	20 μM	Orally, 2 weeks
5.	Silymarin	HFD-induced mice to target on FXR also SIRT1/AMPK activation	420–700 mg/kg	48–50 weeks
6.	Saponin	HFD-induced obese mice to target on SIRT1	25 μM	4 weeks
		HFD induced obese Wistar rats and mice	10 mg/kg	4 weeks
7.	Resveratrol	HFD-induced rats target to Sirt1	200 mg/kg	18 weeks
		HFD-induced rats target to PKA	20 mg/kg	8 weeks
8.	Ursolic acid	HFD-induced obese Mice to target on AMPK pathways	100 mg/kg	15 weeks
9.	Lycopene	HFD-fed mice to target on NF-κB pathway	60 mg/kg	8 weeks
10.	Saponins	HFD-induced mice to target on HCBP6 pathway	25 mg/kg	8 weeks
11.	Dioscin	HFD-induced mice to target on Sirt1/AMPK pathway	80 mg/kg	8 weeks
12.	Glucoraphanin	HFD-fed mice to target on Nrf2 pathway	0.3%	14 weeks

**Table 3:** Treatment of MASLD using nanotechnology.

Herbal Medicine	Nanocarriers	*In vivo*/Clinical Dosing	Reference
Catechin	Polymeric nanoparticles	500 and 1000 μM	[127]
Curcumin	PLGA-NPs	40 mg capsules/day	[128]
Curcumin	PLGA-NPs	40 mg capsules	[129]
Resveratrol	PLGA nanoparticles	500 and 1000 μM	[130]
Chemical synthesis	CeO_2_NPs	10 μg/mL	[131]
Naringenin	Liposome	50–100 mg/kg/day	[132]
*Coptis chinensis*	SLN	50 mg/kg or 0.4 ng/mL	[133,134]

## 2 Results & Discussion

Natural products are a good reservoir of bioactive components such as flavonoids, alkaloids, terpenoids etc., and can serve as a source of constituents to prevent MASLD. Nowadays natural products are gaining interest due to their low cost and minimal side effects.

Natural products may be effective for the treatment of MASLD because of their multiple therapeutic mechanisms. These products have antioxidant, anti-inflammatory, antibiotic, and anti-lipogenic effects, which positively impact the main MASLD progression pathways. For example, curcumin has anti-inflammatory and anti-antioxidant properties grapes contain resveratrol, which reduces hepatic fat accumulation; green tea contains epigallocatechin gallate, which inhibits lipid absorption and enhances fat oxidation; and certain probiotics, prebiotics, and dietary fiber improve the functioning of the gut–liver axis by enhancing intestinal permeability, which decreases liver fibrosis and inflammation. Natural products can be converted into nano-formulations, which protect them from degradation and help in the targeted delivery of drugs; these formulations can also improve the absorption, stability, and solubility of natural products to enhance their therapeutic efficacy.

This review covered various natural products that can be used for the treatment of MASLD, for which no effective therapeutics have been reported; instead, lifestyle modifications are the primary treatment. These natural products contain many phytochemicals that act by specific mechanisms on different pathways to prevent fat accumulation in the liver.

This review not only covered various natural products but also the phytoconstituents they contain, along with the mechanisms through which they exert their effects. Thus, this review may be helpful in the formulation of new therapeutic agents for the treatment of MASLD. Further, this review also suggests approaches to the nanoformulation of natural products, which may enhance their effectiveness.

## 3 Future perspectives

MASLD and its related metabolic illnesses have become increasingly common in recent years because of lifestyle changes. The use of natural products as MASLD therapeutics is a potential new field of research because of the easy availability, efficacy, and low toxicity and side effects of natural products. The overlap in the pathophysiology of metabolic diseases means that MASLD patients may face many other major complications related to the heart, obesity, and diabetes. However, the active ingredients in natural products need to be explored further to determine their active ingredients and any adverse effects and interactions.

## 4 Conclusion

MASLD is the most prevalent liver disease globally and potential treatment options require further investigation. Currently, there are no therapeutics for MASLD; it is instead treated by lifestyle modifications such as diet and physical activity. Herbal or natural products are promising MASLD treatments because of their various phytoconstituents and biological activities. These phytoconstituents act via several mechanisms and can be nano-formulated to improve bioavailability and stability. There are very few side effects of natural products and their natural availability makes them cost-effective. Therefore, herbal treatments should be considered for the treatment of MASLD, for which no therapeutics are currently available.

## Funding

This work received no external funding.

## CRediT authorship contribution statement

**Pooja Yadav:** Formal analysis, Data curation, Conceptualization. **Khushi Quadri:** Methodology, Formal analysis, Conceptualization. **Renu Kadian:** Visualization, Validation, Formal analysis. **Aafrin Waziri:** Visualization, Data curation, Conceptualization. **Pankaj Agrawal:** Resources, Methodology, Conceptualization. **Md Sabir Alam:** Writing – review & editing, Writing – original draft, Visualization, Validation, Supervision, Software, Resources, Project administration, Methodology, Investigation, Formal analysis, Data curation, Conceptualization.

## Acknowledgments

The authors gratefully acknowledge the contributions of their collaborators and co-workers mentioned in the cited references. PY, KQ, and MSA are thankful to the SGT School of Pharmacy and SGT University for their support. AW and PA are thankful to GGSIP University, Dwarka, and RK is thankful to Ram Gopal College of Pharmacy, for their support.

## Declaration of competing interest

The authors declare that they have no conflicts of interest. The final decision on the manuscript and whether or not to publish the findings was made by all of the authors.

## Data availability statement

The data supporting this study’s findings are available on request from the corresponding author. However, the data is not publicly available because of privacy or ethical restrictions**.**

## Ethics statement

No need for ethical approval.

## Informed consent

Not applicable.

## References

[1] Chalasani N., Younossi Z., Lavine J.E. (2012). The diagnosis and management of non-alcoholic fatty liver disease: practice guideline by the American association for the study of liver diseases, American college of gastroenterology, and the American gastroenterological association. Am J Gastroenterol.

[2] Milić S., Lulić D., Štimac D. (2014). Non-alcoholic fatty liver disease and obesity: biochemical, metabolic and clinical presentations. World J Gastroenterol.

[3] Leoni S., Tovoli F., Napoli L. (2018). Current guidelines for the management of non-alcoholic fatty liver disease: a systematic review with comparative analysis. World J Gastroenterol.

[4] Bessone F., Razori M.V., Roma M.G. (2019). Molecular pathways of nonalcoholic fatty liver disease development and progression. Cell Mol Life Sci.

[5] Zhu C., Kim K., Wang X. (2018). Hepatocyte Notch activation induces liver fibrosis in nonalcoholic steatohepatitis. Sci Transl Med.

[6] Younossi Z., Anstee Q.M., Marietti M. (2018). Global burden of NAFLD and NASH: trends, predictions, risk factors and prevention. Nat Rev Gastroenterol Hepatol.

[7] Younossi Z.M., Koenig A.B., Abdelatif D. (2016). Global epidemiology of nonalcoholic fatty liver disease-Meta-analytic assessment of prevalence, incidence, and outcomes. Hepatology.

[8] Wong R.J., Cheung R., Ahmed A. (2014). Nonalcoholic steatohepatitis is the most rapidly growing indication for liver transplantation in patients with hepatocellular carcinoma in the U.S. Hepatology.

[9] Deprince A., Haas J.T., Staels B. (2020). Dysregulated lipid metabolism links NAFLD to cardiovascular disease. Mol Metabol.

[10] Dietrich P., Hellerbrand C. (2014). Non-alcoholic fatty liver disease, obesity and the metabolic syndrome. Best Pract Res Clin Gastroenterol.

[11] Marchesini G., Marzocchi R., Agostini F. (2005). Nonalcoholic fatty liver disease and the metabolic syndrome. Curr Opin Lipidol.

[12] Byrne C.D., Targher G. (2015). MASLD: a multisystem disease. J Hepatol.

[13] Basaranoglu M., Basaranoglu G., Sentürk H. (2013). From fatty liver to fibrosis: a tale of “second hit.”. World J Gastroenterol.

[14] Buzzetti E., Pinzani M., Tsochatzis E.A. (2016). The multiple-hit pathogenesis of non-alcoholic fatty liver disease (NAFLD). Metabolism.

[15] Roeb E., Geier A. (2019). Nonalcoholic steatohepatitis (NASH) - current treatment recommendations and future developments. Z Gastroenterol.

[16] Byrne C.D., Targher G. (2014). Ectopic fat, insulin resistance, and nonalcoholic fatty liver disease: implications for cardiovascular disease. Arterioscler Thromb Vasc Biol.

[17] Hasan K.M., Parveen M., Pena A. (2023). Fatty acid excess dysregulates CARF to initiate the development of hepatic steatosis. Cells.

[18] Takamura T., Misu H., Ota T. (2012). Fatty liver as a consequence and cause of insulin resistance: lessons from type 2 diabetic liver. Endocr J.

[19] Machado M.V., Cortez-Pinto H. (2014). Non-alcoholic fatty liver disease: what the clinician needs to know. World J Gastroenterol.

[20] Heeren J., Scheja L. (2021). Metabolic-associated fatty liver disease and lipoprotein metabolism. Mol Metabol.

[21] Donnelly K.L., Smith C.I., Schwarzenberg S.J. (2005). Sources of fatty acids stored in liver and secreted *via* lipoproteins in patients with nonalcoholic fatty liver disease. J Clin Invest.

[22] Tanase D.M., Gosav E.M., Costea C.F. (2020). The intricate relationship between type 2 diabetes mellitus (T2DM), insulin resistance (IR), and nonalcoholic fatty liver disease (NAFLD). J Diabetes Res.

[23] Zechner R., Kienesberger P.C., Haemmerle G. (2009). Adipose triglyceride lipase and the lipolytic catabolism of cellular fat stores. J Lipid Res.

[24] Brahma M.K., Adam R.C., Pollak N.M. (2014). Fibroblast growth factor 21 is induced upon cardiac stress and alters cardiac lipid homeostasis. J Lipid Res.

[25] Haemmerle G., Lass A., Zimmermann R. (2006). Defective lipolysis and altered energy metabolism in mice lacking adipose triglyceride lipase. Science.

[26] Samuel V.T., Shulman G.I. (2012). Mechanisms for insulin resistance: common threads and missing links. Cell.

[27] Luukkonen P.K., Qadri S., Ahlholm N. (2022). Distinct contributions of metabolic dysfunction and genetic risk factors in the pathogenesis of non-alcoholic fatty liver disease. J Hepatol.

[28] Ferré P., Foufelle F. (2010). Hepatic steatosis: a role for *de novo* lipogenesis and the transcription factor SREBP-1c. Diabetes Obes Metabol.

[29] Song Z., Xiaoli A.M., Yang F. (2018). Regulation and metabolic significance of *De novo* lipogenesis in adipose tissues. Nutrients.

[30] Dong Q., Majumdar G., O'Meally R.N. (2020). Insulin-induced *de novo* lipid synthesis occurs mainly *via* mTOR-dependent regulation of proteostasis of SREBP-1c. Mol Cell Biochem.

[31] Liu L., Yang M., Lin X. (2013). Modulation of hepatic sterol regulatory element-binding protein-1c-mediated gene expression contributes to *Salacia oblonga* root-elicited improvement of fructose-induced fatty liver in rats. J Ethnopharmacol.

[32] Dentin R., Benhamed F., Hainault I. (2006). Liver-specific inhibition of ChREBP improves hepatic steatosis and insulin resistance in ob/ob mice. Diabetes.

[33] Denechaud P.D., Dentin R., Girard J. (2008). Role of ChREBP in hepatic steatosis and insulin resistance. FEBS Lett.

[34] Lawitz E.J., Coste A., Poordad F. (2018). Acetyl-CoA carboxylase inhibitor GS-0976 for 12 weeks reduces hepatic *de novo* lipogenesis and steatosis in patients with nonalcoholic steatohepatitis. Clin Gastroenterol Hepatol.

[35] Fan J.G., Kim S.U., Wong V.W.S. (2017). New trends on obesity and NAFLD in Asia. J Hepatol.

[36] Yki-Järvinen H. (2015). Nutritional modulation of non-alcoholic fatty liver disease and insulin resistance. Nutrients.

[37] Sakurai Y., Kubota N., Yamauchi T. (2021). Role of insulin resistance in MAFLD. Int J Mol Sci.

[38] Basaranoglu M., Basaranoglu G., Sabuncu T. (2013). Fructose as a key player in the development of fatty liver disease. World J Gastroenterol.

[39] Jensen T., Abdelmalek M.F., Sullivan S. (2018). Fructose and sugar: a major mediator of non-alcoholic fatty liver disease. J Hepatol.

[40] Lê K.A., Ith M., Kreis R. (2009). Fructose overconsumption causes dyslipidemia and ectopic lipid deposition in healthy subjects with and without a family history of type 2 diabetes. Am J Clin Nutr.

[41] Tappy L., Lê K.A. (2012). Does fructose consumption contribute to non-alcoholic fatty liver disease?. Clin Res Hepatol Gastroenterol.

[42] Vos M.B., Lavine J.E. (2013). Dietary fructose in nonalcoholic fatty liver disease. Hepatology.

[43] Song Z., Xiaoli A.M., Yang F. (2018). Regulation and metabolic significance of *De novo* lipogenesis in adipose tissues. Nutrients.

[44] Chen Z., Tian R., She Z. (2020). Role of oxidative stress in nonalcoholic fatty liver disease pathogenesis. Free Radic Biol Med.

[45] Begriche K., Igoudjil A., Pessayre D. (2006). Mitochondrial dysfunction in NASH: causes, consequences and possible means to prevent it. Mitochondrion.

[46] Farzanegi P., Dana A., Ebrahimpoor Z. (2019). Mechanisms of beneficial effects of exercise training on non-alcoholic fatty liver disease (NAFLD):roles of oxidative stress and inflammation. Eur J Sport Sci.

[47] Pottoo F.H., Sharma S., Javed M.N. (2020). Lipid-based nanoformulations in the treatment of neurological disorders. Drug Metab Rev.

[48] Raj S., Manchanda R., Bhandari M. (2022). Review on natural bioactive products as radioprotective therapeutics: present and past perspective. Curr Pharmaceut Biotechnol.

[49] Aslam M., Javed M.N., Deeb H.H. (2022). Lipid nanocarriers for neurotherapeutics: introduction, challenges, blood-brain barrier, and promises of delivery approaches. CNS Neurol Disord: Drug Targets.

[50] Kumari N., Daram N., Alam M.S. (2022). Rationalizing the use of polyphenol nano-formulations in the therapy of neurodegenerative diseases. CNS Neurol Disord: Drug Targets.

[51] Javed M.N., Pottoo F.H., Alam M.S. (2016). Metallic nanoparticle alone and/or in combination as novel agent for the treatment of uncontrolled electric conductance related disorders and/or seizure, epilepsy & convulsions. Patent acquired on October..

[52] Mall S.K., Yadav T., Waziri A. (2022). Treatment opportunities with *Fernandoa adenophylla* and recent novel approaches for natural medicinal phytochemicals as a drug delivery system. Explor Med.

[53] Sethiya N.K., Ghiloria N., Srivastav A. (2024). Therapeutic potential of myricetin in the treatment of neurological, neuropsychiatric, and neurodegenerative disorders. CNS Neurol Disord: Drug Targets.

[54] Hooda N., Ahlawat A., Kumari P. (2023). Role of nanomedicine for targeted drug delivery in livestock: FutureProspective. Pharm Nanotechnol.

[55] Kumari P., Shirumalla R.K., Bhalla V. (2024). New emerging aspect of herbal extracts for the treatment of osteoporosis: overview. Curr Rheumatol Rev.

[56] Yadav T., Yadav H.K.S., Raizaday A. (2024). The treatment of psoriasis *via* herbal formulation and nano-polyherbal formulation: a new approach. Bioimpacts.

[57] Yadav R., Yadav T., Upadhayay A. (2024). The influence of phytoconstituents for the management of antipsoriatic activity in various animal models. Antiinflamm Antiallergy Agents Med Chem.

[58] Singh P., Alam M.S., Arif M. (2024). An update on natural bioactive components of traditional preparations for the treatment of nephrolithiasis; a review. J Nephropharmacol.

[59] Kumari P., Devi L., Kadian (2024). Eco-friendly synthesis of *Azadirachta indica*-based metallic nanoparticles for biomedical application & future prospective. Pharm Nanotechnol.

[60] Raza S., Rajak S., Upadhyay A. (2021). Current treatment paradigms and emerging therapies for NAFLD/NASH. Front Biosci.

[61] Teede H., Deeks A., Moran L. (2010). Polycystic ovary syndrome: a complex condition with psychological, reproductive and metabolic manifestations that impacts on health across the lifespan. BMC Med.

[62] Preiss D., Sattar N. (2008). Non-alcoholic fatty liver disease: an overview of prevalence, diagnosis, pathogenesis and treatment considerations. Clin Sci (Lond).

[63] Xu Y., Guo W., Zhang C. (2020). Herbal medicine in the treatment of non-alcoholic fatty liver diseases-efficacy, action mechanism, and clinical application. Front Pharmacol.

[64] Yao H., Qiao Y.J., Zhao Y.L. (2016). Herbal medicines and nonalcoholic fatty liver disease. World J Gastroenterol.

[65] Yang Y., Li W., Liu Y. (2014). Alpha-lipoic acid improves high-fat diet-induced hepatic steatosis by modulating the transcription factors SREBP-1, FoxO1 and Nrf2 *via* the SIRT1/LKB1/AMPK pathway. J Nutr Biochem.

[66] Glasgow E. (2002). Scotland and the encyclopaedia britannica. Libr Rev.

[67] Sahebkar A. (2011). Potential efficacy of ginger as a natural supplement for nonalcoholic fatty liver disease. World J Gastroenterol.

[68] Li Z., Wang Y., Xu Q. (2023). Berberine and health outcomes: an umbrella review. Phytother Res.

[69] Yang S., Cao S., Li C. (2022). Berberrubine, a main metabolite of berberine, alleviates non-alcoholic fatty liver disease *via* modulating glucose and lipid metabolism and restoring gut microbiota. Front Pharmacol.

[70] Vivoli E., Cappon A., Milani S. (2016). NLRP3 inflammasome as a target of berberine in experimental murine liver injury: interference with P2X7 signalling. Clin Sci (Lond).

[71] Zhou H., Feng L., Xu F. (2017). Berberine inhibits palmitate-induced NLRP3 inflammasome activation by triggering autophagy in macrophages: a new mechanism linking berberine to insulin resistance improvement. Biomed Pharmacother.

[72] Zhang D., Liu L., Jia Z. (2016). Flavonoids of Herba Epimedii stimulate osteogenic differentiation and suppress adipogenic differentiation of primary mesenchymal stem cells *via* estrogen receptor pathway. Pharm Biol.

[73] Zhang T.T., Yang L., Jiang J.G. (2015). Bioactive comparison of main components from unripe fruits of *Rubus chingii* Hu and identification of the effective component. Food Funct.

[74] Bouvier F., Rahier A., Camara B. (2005). Biogenesis, molecular regulation and function of plant isoprenoids. Prog Lipid Res.

[75] Grassmann J. (2005). Terpenoids as plant antioxidants. Vitam Horm.

[76] Kim T., Song B., Cho K.S. (2020). Therapeutic potential of volatile terpenes and terpenoids from forests for inflammatory diseases. Int J Mol Sci.

[77] Arendt P., Pollier J., Callewaert N. (2016). Synthetic biology for production of natural and new-to-nature terpenoids in photosynthetic organisms. Plant J.

[78] Choi Y.J., Park S.Y., Kim J.Y. (2013). Combined treatment of betulinic acid, a PTP1B inhibitor, with *Orthosiphon stamineus* extract decreases body weight in high-fat-fed mice. J Med Food.

[79] de Melo C.L., Queiroz M.G.R., Arruda Filho A.C.V. (2009). Betulinic acid, a natural pentacyclic triterpenoid, prevents abdominal fat accumulation in mice fed a high-fat diet. J Agric Food Chem.

[80] Ruan S., Han C., Sheng Y. (2021). Antcin A alleviates pyroptosis and inflammatory response in Kupffercells of non-alcoholic fatty liver disease by targeting NLRP3. Int Immunopharm.

[81] Stegelmeier B.L., Brown A.W., Welch K.D. (2015). Safety concerns of herbal products and traditional Chinese herbal medicines: dehydropyrrolizidine alkaloids and aristolochic acid. J Appl Toxicol.

[82] Kukula-Koch W., Koch W., Angelis A. (2016). Application of pH-zone refining hydrostatic countercurrent chromatography (hCCC) for the recovery of antioxidant phenolics and the isolation of alkaloids from Siberian barberry herb. Food Chem.

[83] Zhang J., Cao H., Zhang B. (2013). Berberine potently attenuates intestinal polyps growth in ApcMin mice and familial adenomatous polyposis patients through inhibition of Wnt signalling. J Cell Mol Med.

[84] Zhang H., Wei J., Xue R. (2010). Berberine lowers blood glucose in type 2 diabetes mellitus patients through increasing insulin receptor expression. Metabolism.

[85] Zhou L., Wang X., Yang Y. (2011). Berberine attenuates cAMP-induced lipolysis *via* reducing the inhibition of phosphodiesterase in 3T3-L1 adipocytes. Biochim Biophys Acta.

[86] Kong W., Wei J., Abidi P. (2004). Berberine is a novel cholesterol-lowering drug working through a unique mechanism distinct from statins. Nat Med.

[87] Kennedy O.J., Roderick P., Poole R. (2016). Coffee, caffeine and non-alcoholic fatty liver disease?. Therap Adv Gastroenterol.

[88] Shokouh P., Jeppesen P.B., Hermansen K. (2017). A combination of coffee compounds shows insulin-sensitizing and hepatoprotective effects in a rat model of diet-induced metabolic syndrome. Nutrients.

[89] Farzaei M.H., Zobeiri M., Parvizi F. (2018). Curcumin in liver diseases: a systematic review of the cellular mechanisms of oxidative stress and clinical perspective. Nutrients.

[90] Cacciapuoti F., Scognamiglio A., Palumbo R. (2013). Silymarin in non alcoholic fatty liver disease. World J Hepatol.

[91] Ou Q., Weng Y., Wang S. (2018). Silybin alleviates hepatic steatosis and fibrosis in NASH mice by inhibiting oxidative stress and involvement with the nf-κB pathway. Dig Dis Sci.

[92] Salamone F., Galvano F., Cappello F. (2012). Silibinin modulates lipid homeostasis and inhibits nuclear factor kappa B activation in experimental nonalcoholic steatohepatitis. Transl Res.

[93] Ni X., Wang H. (2016). Silymarin attenuated hepatic steatosis through regulation of lipid metabolism and oxidative stress in a mouse model of nonalcoholic fatty liver disease (NAFLD). Am J Transl Res.

[94] Li J., Wang R.F., Yang L. (2015). Structure and biological action on cardiovascular systems of saponins from *Panax notoginseng*. Zhongguo Zhongyao Zazhi.

[95] Liu X., Yu J.L., Liu M. (2015). Research progress of bioactivity of steroidal saponins in recent ten years. Zhongguo Zhongyao Zazhi.

[96] Zhang Z.Y., Wu J.P., Gao B.B. (2016). Two new 28-nor-oleanane-type triterpene saponins from roots of *Camellia oleifera* and their cytotoxic activity. J Asian Nat Prod Res.

[97] Yin L.H., Xu L.N., Wang X.N. (2010). An economical method for isolation of dioscin from *Dioscorea nipponica* makino by HSCCC coupled with ELSD, and a computer-aided UNIFAC mathematical model. Chromatographia.

[98] Li M., Han X., Yu B. (2003). Synthesis of monomethylated dioscin derivatives and their antitumor activities. Carbohydr Res.

[99] Liu M., Xu L., Yin L. (2015). Potent effects of dioscin against obesity in mice. Sci Rep.

[100] Shen L., Xiong Y., Wang D.Q.H. (2013). Ginsenoside Rb1 reduces fatty liver by activating AMP-activated protein kinase in obese rats. J Lipid Res.

[101] Yao H., Qiao Y.J., Zhao Y.L. (2016). Herbal medicines and nonalcoholic fatty liver disease. World J Gastroenterol.

[102] Charytoniuk T., Drygalski K., Konstantynowicz-Nowicka K. (2017). Alternative treatment methods attenuate the development of NAFLD: a review of resveratrol molecular mechanisms and clinical trials. Nutrition.

[103] Poulsen M.M., Vestergaard P.F., Clasen B.F. (2013). High-dose resveratrol supplementation in obese men: an investigator-initiated, randomized, placebo-controlled clinical trial of substrate metabolism, insulin sensitivity, and body composition. Diabetes.

[104] Yoshino J., Conte C., Fontana L. (2012). Resveratrol supplementation does not improve metabolic function in nonobese women with normal glucose tolerance. Cell Metabol.

[105] Madak-Erdogan Z., Gong P., Zhao Y.C. (2016). Dietary licorice root supplementation reduces diet-induced weight gain, lipid deposition, and hepatic steatosis in ovariectomized mice without stimulating reproductive tissues and mammary gland. Mol Nutr Food Res.

[106] Wang C., Duan X., Sun X. (2016). Protective effects of glycyrrhizic acid from edible botanical *Glycyrrhiza glabra* against non-alcoholic steatohepatitis in mice. Food Funct.

[107] Hajiaghamohammadi A.A., Ziaee A., Samimi R. (2012). The efficacy of licorice root extract in decreasing transaminase activities in non-alcoholic fatty liver disease: a randomized controlled clinical trial. Phytother Res.

[108] Chen T., Zhong F.J., Hong Y.M. (2014). Effect of *Trifolium pratense* extract on methionine-choline-deficient diet-induced steatohepatitis in C57BL/6 mice. Chin J Nat Med.

[109] Weidner C., Wowro S.J., Rousseau M. (2013). Antidiabetic effects of chamomile flowers extract in obese mice through transcriptional stimulation of nutrient sensors of the peroxisome proliferator-activated receptor (PPAR) family. PLoS One.

[110] Miranda-Henriques M.S., Diniz M.D., Araújo M.S. (2014). Ginseng, green tea or fibrate: valid options for nonalcoholic steatohepatitis prevention?. Arq Gastroenterol.

[111] Hong S.H., Suk K.T., Choi S.H. (2013). Anti-oxidant and natural killer cell activity of Korean red ginseng (*Panax ginseng*) and urushiol (*Rhus vernicifera* Stokes) on non-alcoholic fatty liver disease of rat. Food Chem Toxicol.

[112] Hong M., Lee Y.H., Kim S. (2016). Anti-inflammatory and antifatigue effect of Korean Red Ginseng in patients with nonalcoholic fatty liver disease. J Ginseng Res.

[113] Kumari P., Shirumalla R.K., Bhalla V. (2024). New emerging aspect of herbal extracts for the treatment of osteoporosis: overview. Curr Rheumatol Rev.

[114] Sethiya N.K., Ghiloria N., Srivastav A. (2024). Therapeutic potential of myricetin in the treatment of neurological, neuropsychiatric, and neurodegenerative disorders. CNS Neurol Disord: Drug Targets.

[115] Singh P., Alam M.S., Arif M. (2024). An update on natural bioactive components of traditional preparations for the treatment of nephrolithiasis; a review. J Nephropharmacol.

[116] Chen X.J., Liu W.J., Wen M.L. (2017). Ameliorative effects of Compound K and ginsenoside Rh1 on non-alcoholic fatty liver disease in rats. Sci Rep.

[117] Yuan D., Xiang T., Huo Y. (2018). Preventive effects of total saponins of *Panax japonicus* on fatty liver fibrosis in mice. Arch Med Sci.

[118] Rafie R., Hosseini S.A., Hajiani E. (2020). Effect of ginger powder supplementation in patients with non-alcoholic fatty liver disease: a randomized clinical trial. Clin Exp Gastroenterol.

[119] Ge Y., Zhang Y., Li R. (2011). Berberine regulated Gck, G6pc, Pck1 and Srebp-1c expression and activated AMP-activated protein kinase in primary rat hepatocytes. Int J Biol Sci.

[120] Koperska A., Wesołek A., Moszak M. (2022). Berberine in non-alcoholic fatty liver disease-a review. Nutrients.

[121] Guo Y., Sun Q., Wang S. (2024). *Corydalis saxicola* Bunting total alkaloids improve NAFLD by suppressing *de novo* lipogenesis through the AMPK-SREBP1 axis. J Ethnopharmacol.

[122] Mu H.N., Zhou Q., Yang R.Y. (2021). Caffeic acid prevents non-alcoholic fatty liver disease induced by a high-fat diet through gut microbiota modulation in mice. Food Res Int.

[123] Perumpail B.J., Li A.A., Iqbal U. (2018). Potential therapeutic benefits of herbs and supplements in patients with NAFLD. Diseases.

[124] Boyraz M., Pirgon Ö., Dündar B. (2015). Long-term treatment with n-3 polyunsaturated fatty acids as a monotherapy in children with nonalcoholic fatty liver disease. J Clin Res Pediatr Endocrinol.

[125] Hwang Y.P., Kim H.G., Choi J.H. (2013). S-allyl cysteine attenuates free fatty acid-induced lipogenesis in human HepG2 cells through activation of the AMP-activated protein kinase-dependent pathway. J Nutr Biochem.

[126] Li J., Sapper T.N., Mah E. (2017). Green tea extract treatment reduces NFκB activation in mice with diet-induced nonalcoholic steatohepatitis by lowering TNFR1 and TLR4 expression and ligand availability. J Nutr Biochem.

[127] Pan M.H., Yang G., Li S. (2017). Combination of *Citrus* polymethoxyflavones, green tea polyphenols, and Lychee extracts suppresses obesity and hepatic steatosis in high-fat diet induced obese mice. Mol Nutr Food Res.

[128] Jeon S.Y., Imm J.Y. (2014). Lipase inhibition and cholesterol-lowering activities of laccase-catalyzed catechin polymers. Food Sci Biotechnol.

[129] Jazayeri-Tehrani S.A., Rezayat S.M., Mansouri S. (2019). Nano-curcumin improves glucose indices, lipids, inflammation, and nesfatin in overweight and obese patients with non-alcoholic fatty liver disease (NAFLD):a double-blind randomized placebo-controlled clinical trial. Nutr Metab.

[130] Jazayeri-Tehrani S.A., Rezayat S.M., Mansouri S. (2017). Efficacy of nanocurcumin supplementation on insulin resistance, lipids, inflammatory factors and nesfatin among obese patients with non-alcoholic fatty liver disease (NAFLD):a trial protocol. BMJ Open.

[131] Wan S., Zhang L., Quan Y. (2018). Resveratrol-loaded PLGA nanoparticles: enhanced stability, solubility and bioactivity of resveratrol for non-alcoholic fatty liver disease therapy. R Soc Open Sci.

[132] Parra-Robert M., Casals E., Massana N. (2019). Beyond the scavenging of reactive oxygen species (ROS):direct effect of cerium oxide nanoparticles in reducing fatty acids content in an *in vitro* model of hepatocellular steatosis. Biomolecules.

[133] Chen C., Jie X., Ou Y. (2017). Nanoliposome improves inhibitory effects of naringenin on nonalcoholic fatty liver disease in mice. Nanomedicine.

[134] Xue M., Yang M.X., Zhang W. (2013). Characterization, pharmacokinetics, and hypoglycemic effect of berberine loaded solid lipid nanoparticles. Int J Nanomed.

